# Dengue Fever in South Korea, 2006–2010

**DOI:** 10.3201/eid1809.111811

**Published:** 2012-09

**Authors:** Ji-Hyuk Park, Dong-Woo Lee

**Affiliations:** Korea Centers for Disease Control and Prevention, Cheongwon-gun, South Korea

**Keywords:** dengue, dengue fever, viruses, epidemiology, South Korea, international travel

**To the Editor:** Dengue fever is an acute, febrile disease caused by a flavivirus and is transmitted by *Aedes* spp. mosquitoes (*1*). South Korea is not considered as a region to which dengue virus is endemic because it is located above 35°N latitude and has an isotherm of 10°C in winter, which potentially limits year-round survival of *Aedes aegypti* mosquitoes (*1*,*2*). Thus, dengue fever was seldom recognized as a public health concern in South Korea. However, the first case of dengue fever in South Korea was reported in 1995 in a woman who had traveled to Sri Lanka (*3*). A second case was found in a sailor who had worked in countries in Africa in 2000 (*4*).

Since 2001, dengue fever has been a notifiable infectious disease in South Korea because of concerns about increasing international travel as a source of infection and because the less efficient potential dengue vector, *Ae. albopictus* mosquitoes, were found in this country. All cases reported through the surveillance system should be complemented by thorough epidemiologic investigations to determine whether a case was imported or originated in South Korea. Thus, we analyzed dengue fever–associated data from the Korea Centers for Disease Control and Prevention.

During 2006–2010, a total of 367 suspected cases were reported by physicians through the National Infectious Disease Surveillance System. IgM ELISA and reverse transcription PCR results identified 324 cases as dengue fever. Investigation of 34 cases could not be completed because some cases were in foreigners and in Korean persons who resided in foreign countries, left South Korea after diagnosis, or could not be reached by the contact information that was provided. Investigation of 290 cases was completed by reviewing medical records and by interviews. Interviews were conducted by provincial and Korea Centers for Disease Control and Prevention Epidemic Intelligence Service officers, who used a standardized investigation form.

All 290 case-patients had a history of international travel before onset of dengue fever symptoms. Destination information was available for all 290 case-patients; 17 countries were identified. Visitors to the Philippines (34.1%) contributed the largest number of cases, followed by visitors to Indonesia, India, Thailand, Vietnam, Cambodia, Laos, Malaysia, Myanmar, Bangladesh, China, East Timor, Maldives, Palau, Sri Lanka, Brazil, and Nigeria (Table). These countries are in areas to which dengue fever is endemic or have reported cases (*1*).

**Table:** Presumptive country of origin of imported dengue fever cases, South Korea, 2006–2010

Region, country	No. (%)
Southeast Asia	242 (83.4)
The Philippines	99 (34.1)
Indonesia	37 (12.8)
Thailand	30 (10.3)
Vietnam	24 (8.3)
Cambodia	17 (5.9)
Laos	6 (2.1)
Malaysia	5 (1.7)
Myanmar	5 (1.7)
East Timor	2 (0.7)
Palau	1 (0.3)
Other	16 (5.5)
Southern Asia	40 (13.8)
India	32 (11.0)
Bangladesh	4 (1.4)
Maldives	2 (0.7)
Sri Lanka	1 (0.3)
Other	1 (0.3)
Eastern Asia	
China	2 (0.7)
Africa	
Nigeria	1 (0.3)
South America	
Brazil	1 (0.3)
Other	4 (1.4)
Total	290 (100)

The time interval between the last day of travel and symptom onset was known for 272 (93.8%) of the 290 case-patients. A total of 271 case-patients had traveled within 14 days before symptom onset, and 89 (32.7%) had symptom onset or were given a diagnosis of dengue fever during travel. Symptoms developed within 7 days after travel in 171 (62.9%) persons and 8–14 days after travel in 11 (4.0%) persons. Mean ± SD duration from the last day of travel to symptom onset among 182 case-patients who had symptom onset after travel was 3.20 ± 2.61 days.

Our results indicate that all investigated case-patients had a history of international travel and times of symptom onset during or after travel but within the incubation period for dengue infection. One case-patient had a time to symptom onset of <34 days. This person was eventually given a diagnosis of infection with Epstein-Barr virus but was tested for dengue virus. Because the incubation period exceeded that for dengue virus incubation, this case was classified as an asymptomatic dengue virus infection and an Epstein-Barr virus infection.

Most dengue cases in South Korea are likely imported, and most presumptive countries from which dengue fever originated are in Southeast and southern Asia. These countries are popular holiday destinations for persons from South Korea. Because of distances, few tourists from South Korea travel to Africa and South America (*5*). China is the most popular destination for travelers from South Korea. However, the proportion of persons who acquired dengue infection in China was low (0.7%) because most persons who traveled to China went to Beijing or Shanghai, not to areas in southern China where dengue epidemics have occurred (*5*,*6*).

We report that all dengue fever cases in South Korea during 2006–2010 were imported by persons who had traveled abroad. Global expansion of dengue virus and an increase in international travelers have increased the likelihood of additional cases of dengue fever. In addition, *Ae. albopictus* mosquitoes have been detected in South Korea and can potentially transmit autochthonous dengue infection, as reported in Croatia, France, and Hawaii, USA (*7*–*9*). Thus, more intensified surveillance and investigations should be focused on dengue transmission by *Ae. albopictus* mosquitoes in South Korea.
